# The human colon: Evidence for degenerative changes during aging and the physiological consequences

**DOI:** 10.1111/nmo.14848

**Published:** 2024-06-17

**Authors:** Nicholas Baidoo, Gareth J. Sanger

**Affiliations:** ^1^ School of Life Sciences University of Westminster London UK; ^2^ Blizard Institute, Barts and The London School of Medicine and Dentistry Queen Mary University of London London UK

**Keywords:** aging, collagen, enteric nervous system, glial cells, human colon, inflammaging, interstitial cells of Cajal, microbiome, neurodegeneration, nociception, senescence

## Abstract

**Background:**

The incidence of constipation increases among the elderly (>65 years), while abdominal pain decreases. Causes include changes in lifestyle (e.g., diet and reduced exercise), disease and medications affecting gastrointestinal functions. Degenerative changes may also occur within the colo‐rectum. However, most evidence is from rodents, animals with relatively high rates of metabolism and accelerated aging, with considerable variation in time course. In humans, cellular and non‐cellular changes in the aging intestine are poorly investigated.

**Purpose:**

To examine all available studies which reported the effects of aging on cellular and tissue functions of human isolated colon, noting the region studied, sex and age of tissue donors and study size. The focus on human colon reflects the ability to access full‐thickness tissue over a wide age range, compared with other gastrointestinal regions. Details are important because of natural human variability.

We found age‐related changes within the muscle, in the enteric and nociceptor innervation, and in the submucosa. Some involve all regions of colon, but the ascending colon appears more vulnerable. Changes can be cell‐ and sublayer‐dependent. Mechanisms are unclear but may include development of “senescent‐like” and associated inflammaging, perhaps associated with increased mucosal permeability to harmful luminal contents.

In summary, reduced nociceptor innervation can explain diminished abdominal pain among the elderly. Degenerative changes within the colon wall may have little impact on symptoms and colonic functions, because of high “functional reserve,” but are likely to facilitate the development of constipation during age‐related challenges (e.g., lifestyle, disease, and medications), now operating against a reduced functional reserve.

## Graphical abstract

Reduced nociceptor innervation can explain diminished abdominal pain among the elderly. Degenerative changes occur within the colon wall, especially ascending colon. Mechanisms may include senescence‐like activity and inflammaging. Constipation is more likely to occur during age‐related challenges affecting functions of the bowel that now have reduced functional capacity.

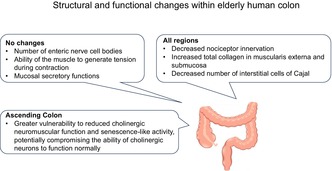

**Key points**

- Reduced nociceptor innervation can explain diminished abdominal pain among the elderly.
- Age‐related changes also occur within the muscle, in the enteric innervation, and in the submucosa. Some involve all regions of colon, but the ascending colon appears more vulnerable. Changes can be cell‐ and sublayer‐dependent. Mechanisms may include development of “senescent‐like” and inflammaging states.
- Constipation among the elderly is more likely to occur during age‐related challenges (e.g., lifestyle, disease, and medications) affecting functions of the bowel that now have reduced functional capacity caused by age‐dependent degenerative changes.

## 1 INTRODUCTION

The elderly experience diminished abdominal pain.^1^, ^2^, ^3^, ^4^ Furthermore, the prevalence of lower bowel disorders is increased.^5^ For example, estimates of chronic constipation among elderly within the community are >7% to >42%,^6^, ^7^, ^8^, ^9^, ^10^, ^11^ rising to >50% within nursing homes.^12^, ^13^, ^14^ Chronic constipation is associated with impaired quality of life and complications which if untreated, can lead to fecal impaction, incontinence, bowel perforations, and increased healthcare costs.^15^, ^16^, ^17^, ^18^, ^19^ Fecal incontinence, more common in older adults,^20^, ^21^, ^22^ may encourage institutionalization.^23^, ^24^, ^25^, ^26^

Medications are often used by the elderly, including opioid receptor agonists (pain relief), drugs antagonizing at muscarinic acetylcholine and other receptors (e.g., antidepressants), and antagonists of Cav1 voltage gated calcium channels (raised blood pressure). These may alleviate abdominal pain and/or disrupt GI motility.^27^, ^28^, ^29^, ^30^, ^31^, ^32^ Constipation among the elderly^14^ can also be associated with disease (e.g., clinical depression, hypothyroidism, and long‐term survival of colorectal/anal carcinoma^18^, ^33^), and changed lifestyle (e.g., reduced calorie and fluid intake and impaired mobility^34^, ^35^, ^36^). The existence and influence of age‐related degeneration on human bowel functions is less clear.

Studies on physiological changes in bowel functions among the elderly provide inconsistent conclusions. In healthy volunteers, age‐related impairment of rectal sensitivity to mechanical distension is reported by some, without changed muscle compliance and tone,^37^ whereas others found no changes.^38^, ^39^ Most studies suggest small and large intestinal motility is well‐preserved during normal adult working lives,^40^, ^41^ but evidence for declined functions among the healthy elderly is inconsistent. For example, movements of the small and/or large intestine were no different to younger adults,^38^, ^42^, ^43^, ^44^, ^45^, ^46^, ^47^ whereas reduced contractile activity^43^ and transit within the small intestine,^48^ reduced migrating motor complex activity,^7^, ^49^ increased oro‐caecal transit times,^50^ and reduced colonic or rectosigmoid transit^44^, ^46^, ^47^ are reported.

Perhaps the effects of aging on the lower bowel can be better understood by studying isolated tissues, so cellular functions can be investigated. Laboratory animals are often used, notably mice and rats. These suggest an age‐dependent loss of extrinsic and enteric innervation, reduced ability of muscle to contract, reduced numbers of pacemaker cells (interstitial cells of Cajal; ICCs) and changes in numbers of enteroendocrine and mast cells within the mucosa.^51^, ^52^, ^53^, ^54^, ^55^, ^56^, ^57^, ^58^, ^59^ However, rodents have high rates of aging, high metabolic rates and differ significantly from humans, in gastrointestinal anatomy, neuronal functions, receptor pharmacology and molecular structures.^60^ This is compounded by extensive genetic variation between different strains of laboratory mice,^61^ influencing, in a strain‐dependent manner, how aging affects gastrointestinal innervation and functions.^62^, ^63^ Thus, to understand how aging affects human intestinal functions it is important to investigate the human. We examine the strength and physiological significance of this evidence.

## 2 SCOPE OF REVIEW

The term “elderly” defines people around 65–75 years of age and above,^64^, ^65^ but is influenced by variables affecting biological aging or senescence, including culture, lifestyle, and genetics. Here, we compare between adults of different ages and not adults versus developing juveniles or children.

The focus is on the colon, perhaps the most readily available, “intact” human gastrointestinal tissue (e.g., “macroscopically normal” tissue from patients with bowel cancer, 5–10 cm away from the tumor).^66^ For robust conclusions, different variables must be considered. First, the different regions of colon must be studied separately, given the differences in positioning, functions, embryonic origin, blood supply, extrinsic innervation, length, and gene expression.^67^, ^68^, ^69^, ^70^, ^71^, ^72^, ^73^, ^74^, ^75^, ^76^ Second, the question “what is normal?” must be considered as it is difficult to fully exclude pathological changes. For example, in colonic mucosa, changes in gene expression can occur up to 10 cm from the tumor.^77^ Nevertheless, when using tissues from the same region and removed for the same disorder, it is possible to compare different age groups. Third, individual variations mean that patient details must be recorded and sample sizes large enough to generate meaningful conclusions.^66^ Finally, studies may be influenced by type of surgery, preparation, and storage of tissue.^66^ All this is important when weighing the strengths and weaknesses of data generated from human tissues.

## 3 SENSORY AFFERENT NEURONS

In human ascending and descending/sigmoid colon from patients aged 24–82 years (*n* = 20, removed mostly for bowel cancer; all regions considered together), a significant reduction was observed in the multiunit response to capsaicin and bradykinin (10–11 donors/group, mostly males) but not the single‐unit colonic mesenteric nerve to capsaicin (*n* = 5; confirming a previous study^78^).^79^ These data suggested an age‐dependent loss of afferent innervation, because chemosensitivity of individual neurons was unchanged. Further studies must examine the effects of aging on noxious mechanical stimuli (reduced in mouse colon) and on pelvic afferent nerves which signal pain and need for defecation.^79^

In an earlier study,^80^ the power was diluted by using human ileum, ascending, transverse, descending and sigmoid colon, and rectum, together, removed mostly for bowel cancer but sometimes for inflammatory and other conditions. With increasing age, resting afferent nerve activity (*n* = 19 tissues) and spontaneous burst firing was decreased. In three sigmoid colons (42‐, 55‐, and 67‐year‐old donors) the increase in afferent nerve activity to bradykinin was thought to be blunted by increased age, and in 10 patients, between 50 and 80 years of age, the density of substance P‐immunoreactive nerve fibers within the mucosa, a marker of afferent nerve endings, was reduced with increasing age.

Reduced immunoreactivity for transient receptor potential ankyrin‐1 (TRPA1) and vanilloid‐1 (TRPV1) channels, and reduced TRPA1 gene transcription was found within sigmoid colon biopsies from healthy elderly versus younger adults (respectively 65–75 and 18–40 years, *n* = 48 and 52).^4^ Both channels are expressed by extrinsic neurons (TRPA1 is also elsewhere such as epithelial and enteroendocrine cells) and may functionally interact.^81^ However, others found no change in TRPA1 RNA expression within the mucosa of human ileum (*n* = 5) and colon (*n* = 15, all regions) combined, comparing <65 versus >65 years of age.^80^

### 3.1 Conclusions

Further work is needed to understand the effects of aging on mechano‐sensitive afferent innervation, paying attention to region of colon and study power. Data suggesting that nociceptor innervation decreases with increasing age appears consistent. They are also consistent with studies among the healthy elderly, demonstrating reduced sensitivity to visceral pain (balloon distention in esophagus and rectum^37^, ^82^) and abdominal pain.^1^, ^2^, ^3^, ^4^ Perhaps as a consequence, the prevalence of irritable bowel syndrome (defined in part by occurrence of abdominal pain) is reduced among the elderly, but less welcome, the elderly have reduced ability to sense pain during appendicitis, delaying onset of medical care.^4^, ^79^

## 4 SMOOTH MUSCLE

The thickness of sigmoid colon circular muscle was unchanged when healthy elderly were compared with younger adults (Table 1). However, such measurements do not reflect functions or changes in constituent parts. Recent studies suggest that total collagen content is increased within colon of the elderly (earlier studies found no changes but higher levels of mature cross‐linked collagen, suggesting increased rigidity; Table 1). For example, in ascending colon from the elderly without diverticulitis, increased total collagen was demonstrated in the muscularis externa (especially *taenia coli*) and submucosa (Table 1).^83^, ^84^ Perhaps for some, this increase reflects diverticulosis that has not progressed to diverticulitis, with progression depending on the subtype of collagen and/or degrading matrix metalloproteinases.^85^ Functional studies using colon from patients with diverticulitis, report increased efficacy and potency of ligands causing circular muscle contraction^86^, ^87^ or longitudinal muscle relaxation.^88^ By contrast, in circular muscle of ascending and descending colon from 15 adult and 19 elderly patients (Table 1),^68^ no differences were found in muscle tension generated during contraction or relaxation evoked by different ligands. The latter appears to conflict with an earlier study using sigmoid colon circular muscle, in which differences were found in amplitude of contractions evoked by different stimuli, between adult and elderly males and females.^89^ However, poor definition of *n*‐values (muscle strips, not patients),^68^ compromises interpretation.

**TABLE 1:** Effect of old age on muscle thickness, collagen content, and contractile ability in the human colon.

Region	Ages/Patients Studied	Observation	Comment
Muscle thickness, collagen content			
Sigmoid colon^110^	18 donors aged between 21 and 94 years	No clear change in thickness of circular muscle	
Ascending, transverse, descending and sigmoid colon^172^	11 and 9 patients aged, respectively, <60 and >60 years	No age‐related changes in total collagen content and no differences between these patients and 5 others with diverticulosis (aged 67–80 years); a higher level of mature cross‐linked collagen in colons from subjects >60 years compared with those <60 years, suggested an increase in rigidity	Assumed full‐thickness tissues. Collagen concentration assessed by measurement of hydroxyproline content
Ascending colon^83^	Adult (22–60 years; 6 males, 6 females) and elderly (70–91 years; 6 males, 4 females)	Histochemical staining demonstrated an increase in total collagen content in submucosa and muscularis externa	Overall increase in collagen concentration assessed by measurement of hydroxyproline content^173^
Ascending and Descending colon^84^	Ascending (adults: 22–60 years; 6 males, 6 females; elderly: 70–91 years; 6 males, 4 females) and Descending (adults: 23–63 years; 6 males, 7 females; elderly: 66–88 years; 6 males, 4 females)	Greater occurrence of total collagen in the *taenia coli* compared with circular muscle	The ascending colon has a greater collagen concentration than the descending colon, as assessed by histochemical and biochemical methods^173^
Muscle contractile ability			
Ascending and descending colon; circular muscle^68^	Adult versus elderly (for carbachol study, respectively, *n* = 7/7 and 8/12 for ascending and descending colon)	No change in tension developed during contraction evoked by carbachol, or during muscle relaxation in response to the nitric oxide donor sodium nitroprusside	Largest study to date
Sigmoid colon circular muscle^88^	~60 years versus mid‐late 70 s	Elderly females more sensitive to carbachol‐induced muscle contraction but elderly males more sensitive to electrically evoked cholinergically mediated contractions	*n*‐values are muscle preparations used, not patients
Proximal, distal, sigmoid colon studied together^174^	Average age was 69 ± 3 years	Age‐dependent increase in ability of dissociated muscle cells to contract in response to different ligands, including carbachol	Perhaps enzyme digestion influences how cells respond

In one other study, collagen fibrils within the submucosa of descending colon became smaller and more tightly packed with increasing age (>60 years; *n* = 7), relative to ascending colon.^90^ A higher collagen content may also exist within the internal anal sphincter of aging incontinent patients (mean 51.5 years),^91^ possibly contributing to reduced ability of the sphincter to maintain resting pressure and achieve maximum squeeze pressure,^92^, ^93^, ^94^ although not all agree.^38^, ^39^, ^95^ In addition to increased collagen content, suggested causes are relative reductions in smooth muscle cells and increased connective tissue with age.^96^, ^97^

### 4.1 Conclusions

The apparent mismatch between muscle function (no differences in tension generated during contraction/relaxation) and structure (increased total collagen) of human colon requires investigation. How does aging affect:

- Different types of collagen in different sublayers of the colon wall.
- The distribution of elastin within the muscularis externa (a preliminary study found increased elastin and collagen around the myenteric plexus of the elderly^98^).
- The ability of the *taenia coli* to contract.
- The ability of *taenia coli* and circular muscle to respond to muscle stretch or distension?

## 5 INTERSTITIAL CELLS OF CAJAL

In human colon, different networks of ICCs orchestrate muscle movements, driven by enteric and extrinsic neurons, and interacting with platelet‐derived growth factor receptor‐positive fibroblast‐like cells, fibroblasts, and glial cells.^99^, ^100^ One group of ICCs is associated with the myenteric plexus. This is stimulus‐dependent (e.g., neuronal), orchestrating high‐amplitude propagating pressure waves. Another is associated with the submuscular plexus and by spontaneously and rhythmically depolarizing (~3 cycles/min), serves as the dominant pacemaker. A third group, within the longitudinal and circular muscles, transmits pacemaker activity throughout the musculature and mediates neural stimuli. Finally, an ICC network exists in association with the subserosa, possibly pacing the longitudinal muscle.

The effects of aging on each network of ICCs within human colon has not been conducted. However, the number and network volume of ICCs within the myenteric plexus and circular muscle have been shown to decrease with age, by ~13%/decade over 25–70 years; the decline was similar in ascending and sigmoid colon and not associated with sex.^101^ Recently, a small study used western blotting to show reduced c‐kit and connexin‐43 protein (markers of, respectively, ICC and gap junction proteins) in the colon (region and muscle layer not specified) of elderly people (*n* = 4 each for 27–36 and 72–82‐year‐old groups).^102^ Perhaps loss of ICCs during aging is related to increased oxidative stress‐inducing cellular apoptosis, combined with an inability to replenish ICCs via stem cells.^101^

### 5.1 Conclusions

In mice, an age‐dependent loss of ICCs uniformly affected all stomach regions and layers (myenteric and muscle layers), unlike disease which might affect discrete areas.^103^, ^104^ For human colon the effect of aging on the different networks of ICCs has not been systematically examined and the consequences on propulsive/retropulsive/segmental movements are unclear. However, since the tissues used were from patients with no reported motility disorders, perhaps the observed changes simply make the elderly more susceptible to other disruptive influences (e.g., lifestyle/medications/disease); this idea is discussed later. By contrast, in sigmoid colon from patients with slow transit constipation (STC), loss of ICCs (associated with myenteric and submucosal plexuses, longitudinal, and circular muscle) and myenteric neurons, has been reported, compared with patients without STC (respectively aged 42–76 and 23–71 years, *n* = 8/6).^105^

## 6 ENTERIC NERVOUS SYSTEM (ENS)

Several animal studies report losses of enteric neurons during aging (e.g., mouse^102^). However, when the accompanying increase in length and thickness of the muscle are considered (changing density of neurons), no age‐related changes were found (mice^106^). In human colon, early reports of loss of enteric neurons, based largely on small studies, are now contradicted by larger studies which found little‐or‐no loss (see below and Table 2). For studies on enteric neuronal numbers in other GI regions (human, animal).^107^, ^108^

**TABLE 2:** Effect of old age on the numbers and densities of enteric neurons and subpopulations in the human colon.

Region	Patients Studied/ages	Enteric nerve populations	Plexus/sublayer	Age‐related change
Ascending, transverse, descending, sigmoid colon^98^	No known digestive pathologies; separated into 20–35 and >65‐year groups, *n* = 6 each; sex not stated	Total (Giemsa stain)/area	Myenteric plexus	Mean 37% loss with increasing age; no differences between regions
Colon; regions not specified^112^	Colon removed for different disorders; 168 males and females; 10 days to 92 years	Different stains used, including NADPH‐diaphorase (NADPH‐d) as a marker of neurons expressing nitric oxide synthase	Myenteric plexus	When all ages considered together, the proportion of ganglia containing empty spaces increased with age, associated with increased surface area of the ganglia, without change in number of NADPH‐diaphorase‐positive neurons/ganglion
Descending & sigmoid colon^113^	Removed for bowel cancer (one unknown); 9 males, 7 females; 33–99 years	Total neuron population (HuC/D, PGP 9.5) and neurons staining for ChAT and nNOS	Myenteric and submucosal plexus, and circular muscle	Myenteric plexus: Loss of HuC/D‐ and ChAT‐IR neuron numbers with age; no change in number of nNOS‐IR neurons Submucosal plexus: No age‐related change in number of HuC/D‐IR, ChAT or nNOS‐IR neurons Muscle: No age‐related change in volume of PGP 9.5‐IR nerve fibers
All regions of Colon^109^	Removed mostly for bowel cancer but also diverticulitis; 19 males and females; 4 months to 95 years	Laser dissection of myenteric ganglia; studied by qPCR, and whole‐mount staining with β‐nicotinamide adenine dinucleotide phosphate (β‐NADPH) and Senescence β‐galactosidase	Myenteric plexus	Increased gene expression in the elderly for the neurotrophin receptor p75 and a subpopulation of nitric oxide synthase in distal colon (~16.6% loss) No age‐dependent change in ChAT gene expression in distal colon but expression smaller in elderly proximal, compared with distal colon
Ascending & descending colon^68^	Removed for non‐obstructive bowel cancer; 36 males and females separated into 35–60 and ≥70‐year groups	Total neuron population (HuC/D, PGP 9.5) and neurons staining for ChAT and nNOS	Myenteric plexus and circular muscle	Numbers of HuC/D and NOS‐IR enteric neurons unchanged with age ChAT‐IR neurons increased in elderly ascending colon but unchanged in descending colon Density of PGP9.5 staining unchanged in ascending colon of the elderly; in descending colon the density was reduced only in deep circular muscle of the elderly
Ascending & descending colon^118^	Removed for non‐obstructive bowel cancer; 48 males and females separated into 22–60 and ≥70‐year groups	Calretinin‐IR enteric neurons	Mucosa	Density of calretinin‐IR enteric neuronal fibers in the ascending and descending colon were reduced in the elderly

Abbreviations: ChAT, choline acetyltransferase; IR, immunoreactive; NADPH, nicotinamide adenine dinucleotide phosphate; nNOS, neuronal nitric oxide synthase; NOS, nitric oxide synthase; PGP9.5, protein gene product 9.5; qPCR, quantitative polymerase chain reaction.

### 6.1 Myenteric plexus

In the largest study conducted (30,306 neurons within ~36 mm of myenteric plexus/antibody/patient from ascending colon of 8 adult and 9 elderly (≥70 years) and 9/10 adult/elderly descending colon), no age‐related differences were found in the number of myenteric nerve cell bodies staining for the pan‐neuronal marker, anti‐HuC/D (anti‐human neuronal protein C/D; labelling cell nucleus and perikaryon), within ascending or descending colon. Furthermore, there was no age‐related difference in numbers of neurons expressing nNOS, nor a decline in neurite extension within the musculature of either region.^68^ However, the numbers of myenteric cell bodies exhibiting ChAT immunoreactivity was increased in ascending colon from the elderly, but not descending colon. Curiously, in the same study, and same area of colon, this change was accompanied by decreased cholinergic neuromuscular function (discussed in Section 7).

A study using laser‐dissected myenteric plexus ganglia (50 ganglia from each donor) to analyze expression of multiple genes (e.g., ion channels, specific neuronal types, senescence, and oxidative stress), found significant differences between children vs. adults (48–58 years) and the elderly (70–95 years).^109^ “Adult” colon (*n* = 4) represented descending or sigmoid colon and “Elderly,” a mix of ascending, transverse, descending, or sigmoid colon (*n* = 11), removed for different disorders. In distal colon, increased gene expression was observed in the elderly for the neurotrophin receptor p75 and for nitric oxide synthase‐1. ChAT gene expression exhibited no age‐dependent differences in distal colon but was lower in elderly proximal, compared with distal colon.

Other studies with large numbers of patients found no age‐dependent changes in several nerve markers within sigmoid colon. In one, nNOS, VIP and SP‐immunoreactive neurons were measured over ages of 21–94 years.^110^ In another, the concentrations of VIP, met^5^‐enkephalin, neuropeptide Y and somatostatin were determined, following extraction from muscle of 28 patients <70 years old and 12 ≥70 years.^111^

A large study using whole‐mount preparations of colon (10 days to 91 years; all regions pooled together) found an age‐dependent increase in proportion of ganglia containing empty spaces.^112^ This was associated with increased surface area of the ganglia. For these ganglia, there was no change in number of NADPH‐diaphorase‐positive neurons/ganglion (nitrergic). Correspondingly, the number of “normal” ganglia (uniformly filled with neurons) declined when all ages were grouped together, but most clearly among the elderly (≥70 years). Others have looked for age‐related changes in the ENS of human ascending and sigmoid colon, testing for linear trends (9 males, 7 females).^113^ A decline in numbers of nerve cell bodies/mm length of myenteric plexus staining for HuC/D, ChAT and PGP‐9.5 was observed, but not nNOS. Analysis as numbers of ganglia/mm length or neurons/ganglia showed similar but less clear trends. However, the regression lines were influenced by a 99‐year‐old patient (tissue removed for unknown reason), separated by 17 years from the next oldest. Confoundingly, the volume of nerve fibers in circular muscle and volume of neuronal structures in myenteric plexus was unchanged with age.^113^

Smaller studies reported a loss of enteric neurons. For example, a 34%–38% decrease in myenteric nerve cells in small intestine and colon (grouped together) comparing 20–35 and >65 years (*n* ≤ 6 each age group),^98^, ^114^, ^115^ accompanied by more numerous collagen and elastic fibers in the ganglia.^98^ Another small study with human colon muscle (layer or region not specified) using western blotting, showed reduced ChAT and nNOS in the elderly (*n* = 4 each for 27–36 and 72–82‐year‐old groups).^102^ Within human small intestine, no age‐dependent changes were found in surface area of enteric ganglia within the duodenum, but the number of neurons was smaller (~16%) within the elderly (65–84 years; *n* = 30 donors but distribution among different age groups not stated).^116^ By contrast, an increased number of NOS‐expressing myenteric neurons were reported in terminal ileum of the elderly (78–86 years; *n* = 8 vs. *n* = 7 younger adults; all with cancer of ascending colon^117^).

### 6.2 Submucosal plexus

A study in which the numbers of myenteric neurons were thought to decline in human colon with increasing age (see above), found no changes in submucosal plexus.^113^

Perhaps specific subpopulations of neurons are vulnerable to age‐related changes. In a preliminary report, the density of calretinin‐immunoreactive neurons and fibers were decreased in the submucosal plexus of ascending colon from the elderly (range of ages: 22–91 years) but not clearly in descending colon. In the mucosa, the decrease in density was greater in ascending versus descending colon.^118^ Others reported colocalization of calretinin‐immunoreactive neurons with vasoactive intestinal peptide.^119^

### 6.3 Conclusions

First, all studies need to be properly powered and small *n*‐values treated with caution. Second, different regions of colon must be studied. Third, aging has little‐or‐no effect on overall numbers of enteric nerve cell bodies in ascending or descending colon, although in the ascending colon myenteric cholinergic neurons may be damaged.

Work is needed to look for possible changes in neuronal subpopulations. A notable omission is the lack of discrimination between cell bodies for enteric motor‐ and inter‐neurons, and intrinsic primary afferent neurons (IPANs). Motor neurons projecting to the muscle appear unaffected by aging (no change in density of neurite extensions within the muscle), but the effects of aging on interneuron varicosities and on IPAN projections into the mucosa (or their functions) are unknown. IPANs detect mechanical and chemical stimuli from the lumen and help initiate propulsive/retropulsive movements.

Finally, the recent identification of age‐dependent changes in lineage composition of the ENS (decline in neural‐crest derived neurons and replacement by mesoderm‐ derived neurons) opens new avenues for research. In mice, the change may be related to loss of enteric neurons. In humans, the consequences are unclear.^120^

## 7 NEUROMUSCULAR FUNCTIONS

A large study concluded that aging impairs cholinergic function in circular muscle of ascending, not descending colon.^68^ Thus, electrically evoked, cholinergically mediated contractions of ascending colon from the elderly were smaller versus younger adults (respectively *n* = 25 and 14), whereas the ability of the muscle to contract in response to acetylcholine was unchanged. The change corresponded with an increase, in the same region of colon, in number of cell bodies staining for ChAT (Section 6.1). The link between these two observations is unclear. However, evidence derived from aging cholinergic neurons of the central nervous system (CNS),^121^ makes it possible to speculate that reduced cholinergic function was related to impaired transport of ChAT to the nerve terminals for synthesis of acetylcholine.^68^ Further work is needed.

Another study found that the amplitude of inhibitory junction potentials in circular muscle of descending colon (evoked by electrical stimulation and largely mediated by ATP from purinergic neurons acting at P_2Y_ receptors^122^, ^123^) declined with increasing age (*n* = 16; 49–84 years) with no change in resting membrane potentials. A decline in women may precede that in men, although the numbers studied (respectively 9 and 7) were small. The physiological consequences are unclear.

### 7.1 Conclusions

Changes in neuromuscular functions during aging may make human ascending colon more susceptible to stool retention. One study examined the distribution of fecal loads and stool retention in 71 patients aged ≥65 years.^124^ The majority (52.1%) with high fecal load scores (significant stool retention) had this within the ascending colon, the remainder being distributed approximately equally between transverse and descending colon and rectosigmoid region.

## 8 NEUROSECRETORY FUNCTIONS

Ussing chamber experiments with human small and large intestine found no age‐related changes in mucosal basal resistance, basal short‐circuit current, or current evoked by neuronal stimulation.^125^ This was a large study (435 donors) using duodenum, jejunum, ileum, ascending, transverse, descending and sigmoid colon, and rectum, obtained mostly from bowel cancer patients but also from other non‐inflammatory disorders. No obvious differences were noted between the different regions or between tissues removed for different disorders, so data were pooled.

### 8.1 Conclusions

The authors^125^ noted that earlier studies on intestinal ion transport in human intestine were small, possessed methodological issues, and had not examined the effects of age. Their findings suggested a lack of age‐dependent changes in basal functions. However, more detailed investigations into neuronal‐mucosal functions are warranted, given the changes observed in neuronal‐muscle functions and the potential for loss of calretinin‐immunoreactive neurons (see Section 6.2).

## 9 ENTERIC GLIAL CELLS (EGC)

EGCs surround myenteric and submucosal nerve cell bodies are within intramuscular layers and mucosa, surround nerve processes and interact with enteroendocrine cells and the epithelial layer.^126^, ^127^ They provide structural, metabolic and trophic support to enteric neurons, participate in neurotransmission, help regulate GI motility,^128^ and provide immunological support and potentially, form new neurons.^129^, ^130^, ^131^, ^132^ Studies into EGCs are complicated by different morphometric and functional characteristics between species, gender, region of gut wall, and by the absence of pan‐glial cell markers.^133^, ^134^, ^135^ In myenteric ganglia of human descending colon, one study found no differences in expression of the gene for the EGCs marker S100 calcium‐binding protein β (*S100β*), between adults (48–58 years) and the elderly (70–95 years).^109^ Another found no differences in expression of *S100β* by muscle layers of human ascending and descending colon from “adult” and “elderly” populations.^136^ By contrast, loss of S‐100β‐immunoreactive EGCs density was reported within the myenteric ganglia and circular muscle of descending colon from the elderly (6 males, 4 females), compared with adult (6 males, 7 females).^137^ In a similar analysis the number of SOX‐10‐immunoreactive EGCs were unchanged.^137^

### 9.1 Conclusions

The functional consequences of reduced S100β‐immunoreactive EGCs within human colon are unclear. Others have associated loss of EGCs with enteric neurodegenerative disorders.^138^ Ablation of EGCs in mice induced changes in neurochemical coding of enteric neurons and altered intestinal motility.^139^, ^140^ However, in human colon the maintained numbers of enteric neurones within the elderly suggests a different function.

## 10 MUCOSAL MAST, ENTEROCHROMAFFIN, AND ENDOCRINE CELLS

A regression analysis suggested that the density of mast cells in mucosa of distal ileum and ascending colon increased over the range of 58–80 years (*n* = 5 and 14 patients respectively), as was the number of enterochromaffin cells in the ileum, but not colon (*n* = 10 and 16 respectively).^80^ With advancing age, mast cells were increasingly found in close apposition to extrinsic nerve terminals suggesting potential compensation for sensory neurodegeneration.^80^ By contrast, in rectal biopsies from subjects below and above 55 years (*n* = 20 each), the mast cell count was reduced in the more elderly, whereas there was no change in numbers of enteroendocrine cells containing 5‐HT and peptide YY.^141^

### 10.1 Conclusions

Further studies are required, which must consider potential region‐dependent differences.

## 11 PATHWAYS OF CHANGE

These are well‐reviewed.^59^ Here, brief summaries are provided, highlighting where human intestine was used.

### 11.1 Inflammaging

Chronic, low‐level inflammation occurs in many tissues with advancing age, including the gut.^142^ This is often called inflammaging. A possible driver is continuous stimulation of macrophages by imbalanced production and clearance of “molecular waste” during aging. This is debris from dead/damaged cells and organelles (including the intestinal microbiome), leading to inflammation when detected by pattern recognition receptors.^143^ Cytokines such as interleukin (IL)‐6, IL‐1β, and TNFα are often linked with inflammaging, and the aging phenotype. In mice, an age‐related increase in expression of pro‐inflammatory cytokines was associated with increased incidence of post‐operative ileus.^144^ Age‐related changes in “pro‐inflammatory” status of macrophages have also been associated with increased cytokines and immune cells in the ENS, and increased loss of ganglionic cells.^145^ Any effects of inflammaging on colonic chemosensitive afferent neurons are likely to be blunted by an age‐dependent loss of afferent innervation (Section 3).

### 11.2 Mucosal permeability

Luminal contents are prevented from crossing into the intestinal wall by epithelial cells within the mucosa, sealed by tight junctions (transcellular proteins, including occludins and claudins). The latter prevent pericellular leakage of luminal solutes, microorganisms and their toxins, digestive enzymes, and undigested food. Tight junctions can break down in the elderly.^146^, ^147^, ^148^ Permeability to solutes, but not macromolecules were increased in terminal ileum biopsies from the elderly, accompanied by elevated expression of IL‐6 which may modulate claudin‐2 expression and solute permeability in the epithelium.^149^ Studies on different regions of human colon are now needed. However, a note of caution is provided by Valentini et al,^150^ who did not observe increased permeability of the small intestine of the elderly in vivo (215 non‐smoking healthy male/female adults, 84 aged between 60 and 82 years), suggesting that low grade inflammation together with relatively minor disease such as Type 2 diabetes, are needed to significantly increase permeability.

### 11.3 Microbiome

The gut microbiota, which differs between ascending and descending colon,^151^ is an important modulator in inflammaging.^152^ The colon has the densest population and richest diversity of microorganisms.^153^ Older people have reduced diversity in microbiota species and phyla.^154^, ^155^, ^156^, ^157^ For example, a genomic study demonstrated loss of bacteria genes involved in producing short chain fatty acids (SCFAs) via fermenting dietary polysaccharides.^154^ SFCAs are an energy source for the microbiota and intestinal epithelial cells, with regulatory and signaling functions in the gut (e.g., increasing mucus production by goblet cells, enhancing intestinal barrier integrity, anti‐inflammatory activity).^158^ Reduced SCFAs can therefore promote gut inflammation.^154^, ^159^ In addition, other bacteria flourish in an inflammatory environment to release effectors which help sustain inflammation.^160^

### 11.4 Oxidative stress and senescence

Aging is associated with reduced autophagy and mitophagy, and increased oxidative stress, which can increase inflammation.^161^ This combination can induce senescence. Senescence is characterized by exit from the cell cycle, and a senescence‐associated secretory phenotype, which includes cytokines and pro‐inflammatory agents.^162^ Senescent cells are normally rapidly cleared by the immune system. However, with increasing age clearance becomes less efficient. Senescent cells remain, enter a state of chronic senescence, continue to secrete pro‐inflammatory molecules, and contribute to inflammaging and the aging phenotype.^163^, ^164^ An increased expression of the chronic senescence marker, *CDKN2A*,^165^ was recently identified in the muscularis externa of ascending and descending colon of the elderly.^136^ Small upregulations of expression of several other genes were also identified, which in ascending colon were more positively associated with increased *CDKN2A* expression than with temporal age. These included genes involved with inflammation, oxidative stress, autophagy, axonal transport, and apoptosis.

Immunofluorescence for p16, encoded by *CDKN2A*, showed strong staining within the cytoplasm of enteric neurons of ascending, not descending colon (5710 neurons examined in 52 “adult” [30–60 years] and “elderly” [70+ years] patients)^136^; these data coincide with reduced cholinergic function in this area of colon.^68^ Interestingly, minimal p16 co‐expression occurred within enteric glial cells (stained by S100β). In summary, these data were surprising for two reasons. Firstly, unlike proliferative glial cells, enteric neurons are post‐mitotic. Secondly, as a cell cycle regulator, p16 expression is usually found within the cell nucleus, not cytoplasm. Nevertheless, precedent exists within other cell types, with suggested roles in non‐cell cycle functions (e.g., protection against DNA damage^136^). The causes of this “senescence‐like” activity are unclear but could be related at least partly to inflammaging and oxidative stress pathways. Perhaps, since enteric neurons are post‐mitotic, cellular debris cannot be “diluted” into daughter cells during mitosis, promoting accumulation of lipofuscin and a senescence‐like state.^166^

### 11.5 Interactions between the endoplasmic reticulum and mitochondria

Within the CNS, such interactions help regulate neuronal functions and changes are linked with neurodegenerative disorders. A recent study described similar interactions and changes within the colonic ENS of healthy and a senescence‐accelerated strain of mouse.^167^ Implications for the human bowel must now be explored.

## 12 CONCLUSIONS

**FIGURE 1:**
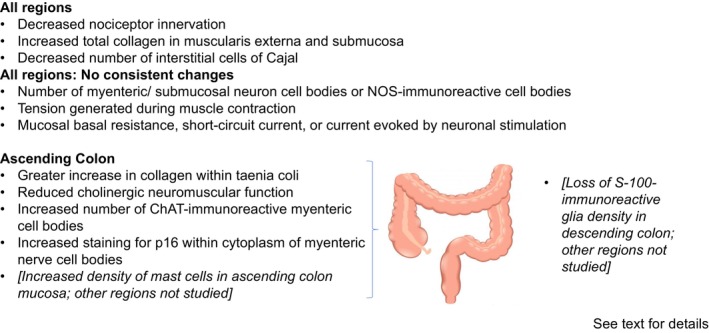
Summary of major structural and functional changes within elderly human colon.

Age‐associated changes in structure and functions of the colon are summarized in Figure 1. These include a decline in myenteric cholinergic neuromuscular function in ascending colon, perhaps caused by dysfunctional nerve axon transport, and expression of the chronic senescence marker p16 within nerve cell cytoplasm. Other changes, in both regions of colon, include reduced numbers of ICCs, increased collagen within the muscle and submucosa, and a decline in nociceptive function. Reduced numbers of EGCs (S100‐immunoreactive) have been observed in descending colon (other regions not studied). What does not change are the total numbers of myenteric and submucosal neurons, the ability of circular muscle to contract and relax, and ability of the mucosa to generate current.

### 12.1 Aging and abdominal pain

Loss of nociceptive afferent innervation of human colon may explain why healthy elderly people have reduced sensitivity to visceral pain.

### 12.2 Region‐dependent aging within the colon wall

Age‐dependent changes can occur throughout the colon (e.g., loss of ICCs), but there is greater vulnerability of myenteric (cholinergic) and submucosal (calretinin‐immunoreactive neurons) innervation within the ascending colon (Table 3). The causes are unclear. Speculation involves loss of epithelial barrier function, allowing harmful material to enter.

**TABLE 3:** Region‐dependent vulnerabilities of the human colon during aging.

Observation	Potential functional consequence
Laser‐dissected myenteric plexus ganglia: Reduced expression of *ChAT* and greater expression of *Ret receptor*, in aging human proximal colon, compared to distal^109^	May reflect reduced cholinergic function in this area of colon, somehow linked with increased ChAT protein staining within nerve cell bodies and compromised transport to nerve terminals^68^
Aging of the human colon significantly increases the risk of stool retention in ascending colon compared with descending colon^124^	Provides insight into vulnerability of aging human ascending colon
Although there was no age‐related change in number of myenteric nerve cell bodies staining for the pan‐neuronal marker, anti‐HuC/D, within ascending or descending colon, an increase in ChAT immuno‐positive myenteric nerve cell bodies was observed in ascending, not descending colon. The latter was linked with a reduced cholinergic neuromuscular function in elderly ascending, but not descending colon^68^	Suggests age‐dependent region‐specific loss of cholinergic function. Hypothesized age‐dependent decrease in axon transport of ChAT from the cell bodies to the cholinergic nerve terminals and hence, a loss of function
Increased staining for p16, a marker of chronic senescence, within the cytoplasm of myenteric nerve cell bodies in aging ascending but not descending colon^136^	Suggests a region‐dependent, post‐mitotic cellular senescence‐like activity involved with aging of enteric neurons
Total collagen content and concentration higher within muscularis externa of the ascending and descending colon from the elderly, but within the *taenia coli* this was higher in the elderly ascending compared with descending colon^83^, ^84^	Suggests a loss of tensile strength for the muscularis externa of all regions of the elderly human colon, but a greater vulnerability of the tenia within the ascending colon, compared with descending colon
Age‐related change in the loss of calretinin‐immunoreactive enteric neurons in the submucosal plexus of the ascending colon, but not clearly in descending colon^118^	Indicates a need to examine the effects of age on specific enteric nerve populations, and in particular, on enteric intrinsic primary afferent neurons, about which nothing is known

Abbreviations: anti‐HuC/D, anti‐human neuronal protein C/D; ChAT, choline acetyltransferase.

### 12.3 Functional reserve

A high reserve capacity of the ENS is suggested for laboratory animals.^52^ This means that the ENS tolerates some degeneration without generating symptoms. A similar physiology is suggested in humans.^68^, ^101^, ^168^ For example, age‐related degenerative changes are identified in “macroscopically normal” colon from patients with non‐obstructive bowel cancer, but these patients were not diagnosed with chronic constipation or other motility disorders.^68^ Thus, functions may be maintained by the remaining cells.

A reduced functional reserve may, nevertheless, increase the likelihood of achieving a “tipping point,” at which symptoms develop when intestinal functions are reduced by other factors (e.g., medications and disease). Similarly, a small, age‐related denervation of anal sphincter musculature reduces its functional reserve, promoting incontinence when looseness of stool or depression of cerebral function, co‐exist.^169^ Complications during childbirth may be exacerbated by age‐related changes in how the pudendal nerve impacts anal sphincter functions in women.^93^, ^170^ In addition, age‐related changes in intestinal permeability may have little importance until compromised by disease such as Type 2 diabetes.^150^

### 12.4 Gaps

What are the effects of aging on:

- Mechano‐sensitive afferent nerve functions (e.g., low threshold mechanosensitivity).
- The muscle response to stretch, especially for *taenia coli* (not studied).
- The different networks of interstitial cells of Cajal.
- Different neuronal phenotypes of the ENS, especially within the submucosal plexus.
- Mucosal permeability and functions.
- Numbers of mast/endocrine cells in different regions of colon.
- Numbers and activity of enteric primary afferent sensory neurons (not studied).
- Males versus females in sufficiently powered studies.
- Pathways leading to damage.
- Mechanisms which make the ascending colon relatively more vulnerable to change.

Consideration needs to be given to the relationships between changed cellular structures and functions, and the pathophysiology of the intact lower bowel. Many reports a greater incidence of chronic constipation and related symptoms among the elderly (see Introduction) so it seems reasonable to suppose that these are at least partly, caused by degenerated functions. However, a Rome IV analysis highlighted a decline in gut–brain disorders among the elderly, including irritable bowel syndrome, functional dyspepsia and functional constipation.^171^ Perhaps the mismatch is explained by reduced pain sensitivity and by differences between constipation that is “functional” or related to degenerative changes.

## 13 LESSONS

RNA expression does not necessarily translate to changes in protein expression. This is not a new understanding but for the aging colon, it is illustrated by data on S100‐immunoreactive glial cells and the increased staining for p16 within the ascending, not descending colon, predicted by RNA expression to increase in both regions.

Given the natural variation among the human population and “life experiences,” the size of the study matters. For example, small studies claim differences in number of enteric neurons in “adult” and “elderly” populations, but larger studies report little‐or‐no change.

Different regions of colon must be studied separately as each has different functions and potential to age differently.

## AUTHOR CONTRIBUTIONS

NB and GJS drafted the manuscript and contributed substantially to all aspects of the article and revised versions.

## FUNDING INFORMATION

No specific funding was related to the construction of this manuscript. Previously, GJS has been funded for age‐related research by the research into aging fund, set up and managed by AgeUK, by Dunhill Medical Trust for research into Ageing and the Human Bowel (grant number R382/1114), by the Bowel and Cancer research charity and by Takeda Pharmaceuticals.

## CONFLICT OF INTEREST STATEMENT

NB declares no conflict of interest. GJS acts as scientific advisor for BYOMass.

## Data Availability

Data sharing is not applicable to this article as no new data were created or analyzed in this study.
